# Dental periodontal procedures: a systematic review of contamination (splatter, droplets and aerosol) in relation to COVID-19

**DOI:** 10.1038/s41405-021-00070-9

**Published:** 2021-03-24

**Authors:** Ilona G Johnson, Rhiannon J Jones, Jennifer E. Gallagher, William G. Wade, Waraf Al-Yaseen, Mark Robertson, Scott McGregor, Sukriti K. C, Nicola Innes, Rebecca Harris

**Affiliations:** 1 Cardiff University School of Dentistry, Applied Clinical Research and Public Health, College of Biomedical and Life Sciences, Heath Park, Cardiff, UK; 2 Cardiff University School of Dentistry, Dental Education, Scholarship & Innovation, College of Biomedical and Life Sciences, Heath Park, Cardiff, UK; 3 Faculty of Dentistry, Oral & Craniofacial Sciences, King’s College London, Denmark Hill Campus, Bessemer Road, London, UK; 4 King’s College London, London, UK; 5 School of Dentistry, College of Biomedical and Life Sciences, Cardiff University, Heath Park, Cardiff, UK; 6 School of Dentistry, Child Dental and Oral Health, University of Dundee, Dundee, UK; 7 Library and Learning Centre, University of Dundee, Dundee, UK; 8 Faculty of Dentistry, Oral & Craniofacial Surgery, King’s College London, Centre for Host Microbiome Interactions, Denmark Hill Campus, Bessemer Road, London, UK; 9 School of Dentistry, College of Biomedical and Life Sciences, Cardiff University, Heath Park, Cardiff, UK; 10 Department of Public Health, Policy & Systems, University of Liverpool, Room 124, 1st Floor, Block B, Waterhouse Building, Liverpool, UK

**Keywords:** Health care, Infection control in dentistry

## Abstract

**Introduction:**

The emergence of the SARS-CoV-2 virus and subsequent COVID-19 pandemic has had a significant effect on the delivery of routine dentistry; and in particular, periodontal care across the world. This systematic review examines the literature relating to splatter, droplet settle and aerosol for periodontal procedures and forms part of a wider body of research to understand the risk of contamination in relation to periodontal care procedures relevant to COVID-19.

**Methods:**

A search of the literature was carried out using key terms and MeSH words relating to the review questions. Sources included Medline (OVID), Embase (OVID), Cochrane Central Register of Controlled Trials, Scopus, Web of Science and LILACS, ClinicalTrials.Gov. Studies meeting inclusion criteria were screened in duplicate and data extraction was carried out using a template. All studies were assessed for methodological quality and sensitivity. Narrative synthesis was undertaken.

**Results:**

Fifty studies were included in the review with procedures including ultrasonic scaling (*n* = 44), air polishing (*n* = 4), prophylaxis (*n* = 2) and hand scaling (*n* = 3). Outcomes included bacterial (colony-forming units e.g. on settle plates) or blood contamination (e.g. visible splatter) and non bacterial, non blood (e.g. chemiluminescence or coloured dyes) contamination. All studies found contamination at all sites although the contamination associated with hand scaling was very low. Contamination was identified in all of the studies even where suction was used at baseline. Higher power settings created greater contamination. Distribution of contamination varied in relation to operator position and was found on the operator, patient and assistant with higher levels around the head of the operator and the mouth and chest of the patient. Settle was identified 30 min after treatments had finished but returned to background levels when measured at or after an hour. The evidence was generally low to medium quality and likely to underestimate contamination.

**Conclusion:**

Ultrasonic scaling, air polishing and prophylaxis procedures produce contamination (splatter, droplets and aerosol) in the presence of suction, with a small amount of evidence showing droplets taking between 30 min and 1 h to settle. Consideration should be given to infection control, areas of cleaning particularly around the patient and appropriate personal protective equipment, with particular attention to respiratory, facial and body protection for these procedures. In addition, the use of lower power settings should be considered to reduce the amount and spread of contamination.

## Background

The emergence of the novel variant of the severe acute respiratory syndrome–related coronavirus (SARS-CoV-2) and the subsequent global coronavirus disease (COVID-19) pandemic has had a significant impact on oral health systems across the world.^1,2^ Policy makers and clinical teams have had to re-evaluate and adapt dental care delivery and systems in response to the challenges. One of the key issues for dentistry has been that the main mode of transmission of this virus is considered to be through direct contact, droplets and fomites, although there is increasing evidence of aerosol transmission (WHO).^3,4^

Dental care has been delivered for many years in the United Kingdom on the basis of universal (standard) infection control precautions based on the premise that any patient can carry pathogens and potentially transmit infection.^5^ The World Health Organisation has recommended droplet and contact precautions when caring for patients with COVID-19 and airborne precautions during the delivery of aerosol generating procedures (AGPs).^3^ Dental professionals have therefore needed to wear additional respiratory protection, gowns, eye protection,^6–9^ and comply with a range of additional recommended procedures for infection control during the pandemic.^10^

Periodontal disease is very common in the population, with UK surveys finding pockets of 4 mm or more in just under 20% of 16–24 year olds with this figure rising to 82% of 74–84 year olds suggesting significant treatment need within the population.^11^ Periodontal care is routinely provided in primary care and is some of the most common care undertaken in day-to-day dental practice. Estimates from NHS England show that 44.5% of adult courses of treatment include scale and polish procedures.^12^ In order to treat periodontal conditions appropriately in accordance with contemporary guidance/practice, it is important to determine which periodontal procedures produce droplets, and which are likely to generate aerosols. Furthermore, it is important to understand the risks of transmission associated with these activities.

Aerosol has been generally defined in terms of inspirable particles generated by humans and the environment^13^ and is typically defined as a suspension of liquid or solid in air with particle sizes of <5 μm,^14^ although some authors have used a higher size limit including particles up to 50 μm in size.^15,16^ In this review, the former, more restrictive, definition will be used. There is evidence to indicate that once generated, aerosols can remain in the air for many hours^17,18^ requiring time to allow air changes to clear this risk.^19^ Within healthcare, droplets are considered to be inspirable particles larger than 5 μm in diameter which again are deemed to require time to settle onto surfaces before decontamination.^19^ Some papers describe an additional category of larger droplets (over 50 μm in diameter) as splatter:^16,20^ these are a mix of air, water, and/or solid substances which behave in a ballistic or projectile manner visible to the naked eye.^21^

Aerosol generating procedures (AGPs) in dentistry have been defined as: “dental procedures using high speed devices such as ultrasonic scalers and drills”, which provides limited information about droplet and aerosols associated with specific periodontal procedures. Ultrasonic and sonic scaling refers to the use of instruments with high vibrational energy which is conducted to a scaler tip, causing vibrations with frequencies in the range of 25,000–42,000 Hz.^22^ However, whilst ultrasonic scaling is included within definitions of AGPs, there is less clarity with regards to other procedures. Conventional prophylaxis for example involves the use of a mechanical handpiece, rubber–cup and prophylaxis paste, while air polishing is used to remove plaque biofilm and stains and involves the use of a handpiece that generates a slurry of pressurised air, abrasive powder.^23^ Other procedures can also include hand instrumentation and a range of surgical options^24^ - but the extent of splatter, droplets and aerosol associated with these received relatively little attention prior to the emergence of Sars-COV-2 virus and the COVID-19 pandemic.

In the absence of an established evidence base, professional organisations have tried to address uncertainties about the safety of providing clinical dentistry by issuing advice during the pandemic.^25^ Guidance relating to prophylaxis, emphasised that this is recognised as part of professional mechanical plaque removal in people with periodontitis, and if withheld could result in clinical harms to these patients.^24^ Prophylaxis undertaken with a slow handpiece, with no water, reduced prophy paste and due diligence was deemed to be a non-AGP based on emergent particle size,although polishing teeth for purely cosmetic reasons was not recommended.^25^ However, anxieties concerning providing periodontal care appear to remain,as indicated in a recent survey of British Society of Periodontology and Implant Dentistry members which found that the majority had concerns about their ability to provide appropriate levels of care and concerns about infection risks.^26^

Previous studies have reviewed the literature for aerosol generation and contamination in dentistry but these did not look at the specific risks relating to procedures for periodontal care in detail.^27^ To carry out dental treatments using appropriate infection control precautions for COVID-19 and take measures to mitigate for them, it is necessary to understand which periodontal management/treatment/care procedures produce aerosol and droplets, to what extent, and how contamination spreads within the surgery.

The objectives of this review are to

1. Characterise the pattern baseline of splatter, droplet settle and aerosol spread relevant to periodontal procedures (e.g. ultrasonic scaling, air poinshing, hand scaling and prophy with pumice) in the dental surgery
2. Record outcomes and outcome measures in studies of contamination arising from periodontal procedures
3. Explore the influence of procedural delivery on baseline (e.g. power settings) on splatter, droplet settle and aerosol
4. Identify gaps in the evidence.

## Methods

This review was conducted as part of a systematic review registered und**e**r the International Prospective Register of Systematic Reviews (ID number 193058).^28^ Overarching summary findings of this review^29^ and the methods have been reported.^28^ This is the second of a series of detailed analyses of the literature specific to procedural areas. The first paper reported on oral surgery.^30^ This paper presents the analysis of 50 papers identified for periodontal procedures. Papers were identified though a systematic search of key databases (Medline (OVID), Embase (OVID), Cochrane Central Register of Controlled Trials, Scopus, Web of Science and LILACS) and ClinicalTrials.gov. Key words and MeSH (Medical Subject Headings) were used and further details for eligibility criteria are available.^28^ Searches were carried out in May 2020 and updated on 11th August 2020. Citation tracking was undertaken for selected papers (backwards and forwards) to identify any further possible papers. A total of 723 papers were identified after duplicates were removed. Papers were then screened for titles and abstracts by two reviewers from the team (*n* = 8) in Rayyan,^31^ independently and in duplicate, with differences resolved by consensus with a further member of the team. Full text review was carried out by two members of the team. Experimental (including manikins and modelling), observational, trials, qualitative and other relevant studies were included, where there was a measurement of aerosol, droplets or splatter directly linked to periodontal treatment. Studies were excluded where aerosol, droplet or splatter generation were not linked to a single treatment procedure. Data items were extracted via standardised data extraction form (developed, refined and tested for the task) to an excel table by eight trained reviewers. Areas where information was unclear or missing were resolved by contacting study investigators and where this was unsuccessful, consulting with another reviewer. A Preferred Reporting Items for Systematic Reviews and Meta-Analyses flowchart was devised to outline the process.^29^ Data extraction included study demographics, procedures, detection methods (microbiological and non-microbiological) and outcomes. The baseline or control data without mitigation (e.g. mouthwashes or suction) were used for interventional studies which were for the purpose of reducing aerosol or contamination. Quality assessments were carried out for all studies using template tools (Appendix 1) to accommodate the diverse methodologies used in the studies. Sensitivity analysis was also undertaken by an expert in microbiology, considering study methodologies and used a traffic light system for possible over or under reporting of contamination.^29^ These are reported in the main overview paper of this review study.^29^

## Results

A total of 50 papers were identified which examined aerosol relating to periodontal treatment and procedures.^16,20,32–79^ Publication dates ranged from 1969 to 2015 and originated from 15 countries (Brazil *n* = 3; Canada *n* = 1; Finland *n* = 1; Germany *n* = 1; India *n* = 17; Iran *n* = 2; Italy *n* = 1; Japan *n* = 2; Malaya = 1, Netherlands = 1; Republic of Korea *n* = 1; Romania *n* = 2; Taiwan *n* = 1; UK *n* = 1; USA *n* = 15).

Of the periodontal papers, the majority only looked at one type of periodontal procedure. At total of 44 papers examined ultrasonic scaling,^20,32–50,52,53,55–58,60–67,69–72,74–79^ a further four considered air polishing,^51,54,59,68^ two looked at prophylaxis,^16,53^ and three studies involved hand scaling.^16,62,73^ No papers were identified for surgical periodontal procedures. Of these, the majority (*n* = 35) were interventional. All other studies (*n* = 15) were observational. Most (*n* = 42) were conducted in clinical settings in hospitals (*n* = 29) and general practices (*n* = 10). Four studies did not include details of the setting.^35,41,70,79^ Six studies used simulation within a confined environment (a box or a chamber).^16,42,59,60,62,65^

### Device settings and baseline suction

Of the 44 ultrasonic papers only 15 mentioned power or device settings, and only 2 compared differences in the power settings of the ultrasonic units/devices^62,79^ (Appendix 2), while these papers identified increased contamination arising from higher power settings, there was insufficient information and too much heterogenicity for further analysis of this data. Half of the ultrasonic studies used suction at baseline during procedures; one study stated that they did not use suction and the remainder did not provide any information about suction use. Contamination was identified in studies which did, and did not, use suction at baseline.

Device settings were included in one air polishing study report and none of the prophylaxis papers. None reported on the use of suction and none compared power settings.

The studies identified contamination in three main ways: (i) person contamination (operator, assistant or patient); (ii) environmental contamination (surfaces within the dental operatory); and (iii) air contamination (collection of aerosolised contaminants from the air).

i. Person droplet/splatter contamination

**Fig. 1:**
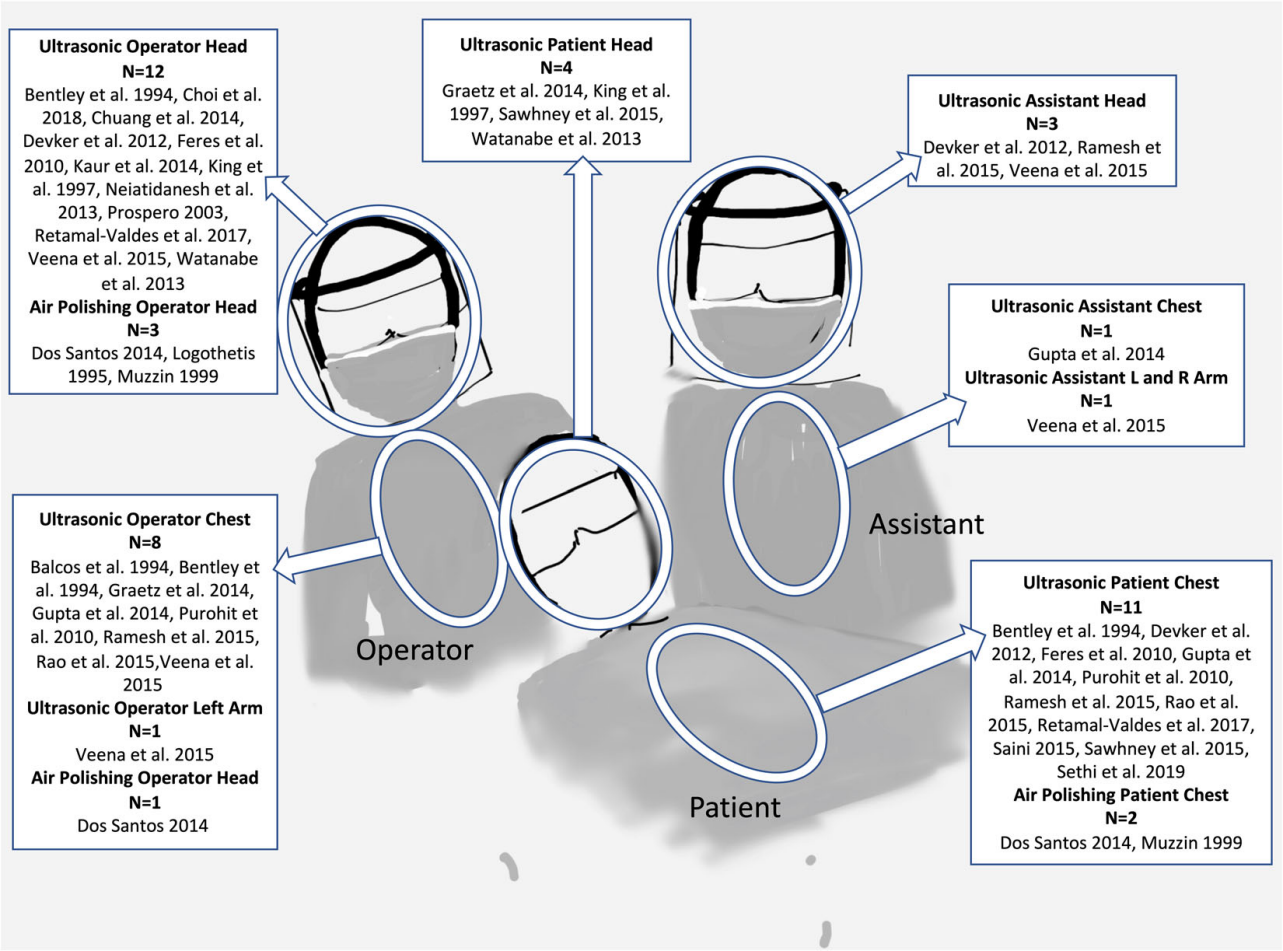
Illustrated overview of person droplet/ splatter contamination studies. This indicates the areas and studies where person contamination was measured.

All person contamination papers identified a positive result in all areas examined. Person contamination was identified in 22 of the 44 ultrasonic papers, three of the four air polishing papers and one of the two hand scaling papers (Fig. 1/Appendix 3). There were 17 microbiological and 5 non-microbiological studies which looked at person contamination (Appendix 4).

#### Person contamination: ultrasonic scaling

Operator Head and neck contamination was identified in 14 ultrasonic papers. Significant contamination was found in most studies and the distribution of contamination extended in the studies that examined at this, to inside the full-face shield^70^ and inside the face mask.^78^ The body (chest and arm of the operator) were also significantly contaminated in eight studies. Where reported, contamination was greater with higher power settings.^79^ Contamination appeared to vary with body position, including being left or right handed.^35,78^

Few papers (*n* = 4) considered at contamination of the assistant, however, all sites that were examined had positive findings. Of these, two studies identified contamination of the assistant’s head, including one that identified contamination under the mask.^78^ Two further studies found contamination of the body (chest and side) of the assistant. Three of the studies examined patient and assistant and all found that the assistant was less contaminated than the operator.

Patient contamination was identified in 14 papers. This included five studies of the head and neck and 11 studies of the patient’s chest, all of which found contamination in each of the areas examined. There was significant heterogeneity between papers in terms of measures and methodologies, which meant that comparisons could not be carried out. Furthermore, there was no data relating to infectivity of contamination.

#### Person contamination: air polishing and hand scaling

All three air polishing papers examined operator facial contamination. Two of the papers also measured patient body contamination (chest area). Of these, one found greater contamination on the patient^51^ and one found greater contamination on the operator.^68^

Operator and assistant face contamination were included as part of the paper that assessed hand scaling however this review did not report on the data findings for this.^73^

ii. Environmental splatter/ droplet contamination and spread

#### Ultrasonic studies: splatter/droplet contamination and spread

**Fig. 2:**
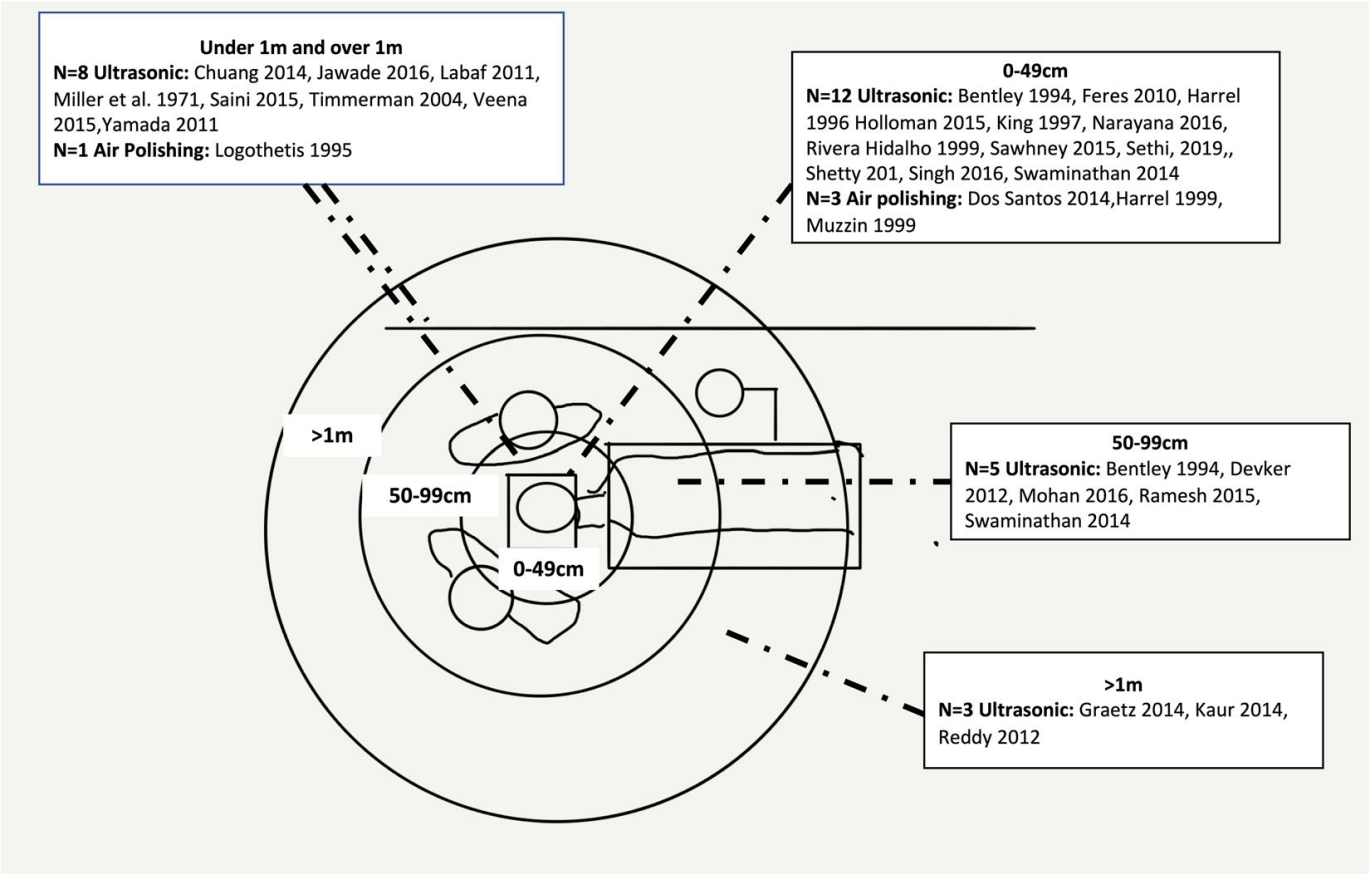
Illustrated overview of environmental droplet/ splatter contamination spread studies. This indicates the studies that measured this and distances from the operating site which were measured.

In total, 26 papers reported environmental splatter/droplet contamination and spread in relation to ultrasonic procedures. All studies identified contamination at the sites sampled (Fig. 2, Appendix 5). Eight papers measured contamination in the environment at both under and over 1 m from the patient and three studies only looked over 1 m. These studies indicated that the greatest amount of contamination was nearest to the patient and contamination decreased with increasing distance from the patient. Research that examined different sites around the dental surgery found that the left side of the patient (when the operator was on the right) and in front of the patient were more contaminated than other areas, for example behind the operator.^40,78^ Contamination was identified at the furthest distance measured in each of the studies. The farthest point measured in the studies was 3.0 m and 2.7 m (9 ft) for ultrasonic^55,57^ and 2.7 m (9 ft) for air polishing in a room with 13 air changes per hour.^54^

Three studies collected data on contamination after ultrasonic treatment was complete.^61,78,80^ One study identified colony-forming units (CFUs) on petri dishes which were opened at the end of treatment and lids were closed 30 min after the end of care.^61^ Of the two remaining studies, one found contamination at sites one foot from the treatment site at 30 min after treatment—but none at 60 min.^78^ A further study found that samples collected 2 h after treatment showed contamination had returned to baseline levels, and reduced further at 4 h.^80^

#### Air polishing, prophylaxis and hand scaling: splatter/droplet contamination and spread

All four air polishing papers that examined environmental splatter/droplet contamination and spread identified contamination at all sites sampled (Fig. 2, Appendix 5). One air polishing paper collected samples at both under and over 1 m from the patient; however, they found greater contamination nearest the patient, behind the dental chair and to the right of the patient.^54^ The air polishing studies only measured contamination relating to the procedure; settle after procedures was not examined. None of the prophylaxis or hand scaling studies considered environmental splatter or droplets.

iii. Air contamination

#### Ultrasonic studies: air contamination

Seven studies examined air samples; these used evacuation/vaccum devices relating to ultrasonic procedures. Two studies reported blood in air samples^72,77^. Of which Barnes,^72^ took samples next to the operating site and showed that blood was aerosolised, but did not report particle size, distance or suspension in the air. The other, Yamada,^67^ took air samples at distances 50 cm and 100 cm away and used visible eye contamination on a filter.

The five studies that examined bacteria in air samples identified aerosolised bacteria. One small study^80^ found that bacterial counts increased 7–34 fold during ultrasonic treatment in a closed surgery which fell by 80% at the completion of treatment. Counts had returned to baseline by 2 h post-treatment. Three of the studies detected bacteria in air samples using microbiological approaches with low sensitivity^64,65,71^ and the remaining ultrasonic study looked specifically for ampicillin resistant streptococci.^66^

#### Air polishing, prophylaxis and hand scaling: air contamination

One study examined both prophylaxis (polishing with pumice) and hand scaling. This was carried out in laboratory conditions and found low numbers of CFUs arising from hand scaling (between 1 and 15 CFU/min, median 1), and higher levels arising from prophylaxis with pumice (between 4 and 270 CFU/min, median 42). None of the air polishing studies included air contamination samples.

## Discussion

This review presents the findings of the literature relating to droplets, splatter and aerosol generated though periodontal treatments. The findings, regardless of study design, demonstrate that all procedures produce some form of contamination (droplet, splatter or aerosol). This review did not set out to examine mitigation of contamination (e.g. the use of suction or mouthrinses) and many studies did not report the use of suction but it is possible that this was used at baseline. However, it was clear that evidence of contamination was present, irrespective of whether dental high or low volume suction was used at baseline.

The amount of contamination varied between studies and there was a small amount of evidence to suggest that the way that instruments were used e.g. with higher power settings, and operator position (e.g. greater contaminaton to the left and in front of a patient with a right handed operator) affected the amount of contamination produced and where it went.

Most of the literature related to ultrasonic scaling, and, concurrent with early reviews of the literature,^15^ showed that this produced a significant amount of contamination. Our review demonstrated that bacteria (and therefore likely viruses) were aerosolised and were transmitted as droplets and splatter which travelled up to 3 m in the surgery. There was also clear evidence of blood and bacteria in air samples. No further distances were tested for contamination in the studies reviewed and therefore the true extent of spread may be greater.

There was limited evidence relating to air polishing, prophylaxis and hand scaling. Studies that looked at air polishing found this produced significant contamination over distance, even where there were more than 10 air changes per hour^54^ as recommended in current infection control literature.^10,81^ Studies relating to polishing of restorations were excluded from the review, leaving one study of prophylaxis which found contamination in the air. Hand scaling produced a small amount of contamination and spread aligning to its categorisation in Dental COVID-19 Standard Operating Procedures as a non-AGP.^82^

The majority of studies measured contamination during or immediately after treatment with only three studies looking specifically at contamination after treatment. Contamination was identified at 30 min after the cessation of ultrasonic treatments^61^ and contamination levels were reported to have returned to baseline when measured at 1 and 2 h after treatment in two studies^78,80^ suggesting that airborne contaminants may remain with ongoing settle of surface fomites at the 30 min, reducing to undetectable levels of settle within an hour.

The highest levels of contamination were closest to the operating site with the patient and operator most susceptible to contamination. Patient contamination was most frequently found around the chest and face. Infection control measures normally suggest the use of eye protection and an apron or body coverage for the patient during treatment. This review supports this recommendation, however, in view of the amount of contamination and its non-visible nature, consideration should be given to careful removal and decontamination or disposal of eye and body protection. No studies investigated contamination below the waist of the patient and it is not possible to determine whether additional protection would be recommended there.

The operator’s head and body were identified as being particularly vulnerable to contamination in the present review. Studies found contamination of the facial area, including masks and visors when these were used, that extended to the inside of personal protective equipment (mask and face shield).^70,78^ As such this review highlights the importance of using effective respiratory protection and personal protective equipment coverage and subsequent safe removal^6,7^ when carrying out periodontal procedures.

There was very little evidence relating to individual pathogens and most microbiological studies measured CFUs with variable (most often low) sensitivity methodology. A range of different methods were used limiting comparability. It was not possible to identify whether or not transmission of bacteria or viruses to dental staff or patients occurred, as this was not reported in any of the studies. There was also very limited evidence relating to environment factors such as the frequency of air changes, use of air conditioning and any potential cumulative effect of real-world sequential treatments. The findings of many of the included studies may underestimate the amount of and spread of droplets, splatter and aerosol due to low sensitivity and confounding factors such as suction and power settings which were poorly reported. Furthermore, most studies only captured samples at specific locations, with the majority of samples being taken at the operator or patient level. Studies lacked data in relation to the wider clinical environment for example the floor, clinical surfaces and air around the surgery and walls making it difficult to determine the extent to which contamination occurs in relation to current infection control guidance.

The emergence of COVID-19 will inevitably generate further research in this area and this will be important to further inform the safety of staff and patients, particularly as it is likely that we will have to live with this virus for some time. One of the challenges for dental teams has been the lack of consolidated and rapidly available evidence to support practice in response to this novel virus. As part of the research efforts to support practice, the findings of the main review of all procedures and proposed categorisations for AGPs^28^ were shared with working groups^83^, to inform action and avoid replication of searches or reports.

This report extends the authors previous work and provides additional detailed analyses of the literature relating to splatter, droplets and aerosol outcomes for periodontal procedures. Further evidence is needed for non-ultrasonic and surgical periodontal procedures. Future studies which use comparable and consistent methodological approaches are recommended to explore the pattern of environmental, person and air contamination in the dental setting from periodontal procedures. These will provide much needed evidence to inform personal protective equipment and infection control guidance in dentistry.

While the present review explores the spread and pattern of droplets, splatter and aerosol, there remains little evidence to indicate the risk of transmission of SARS-CoV-2 and other infectious agents through these routes. In addition, more detail is needed to inform the time for infectious droplets and splatter to settle and airborne contaminants to clear specifically in relation to air changes and other environmental factors for relevant to the management of COVID-19 in dental care.

## Conclusion

Ultrasonic, air polishing and prophylaxis procedures produce contamination (splatter, droplets and aerosol). Contamination is generated in the presence of suction with the highest levels around the patient, with location depending on operator handedness and position. Hand scaling produces minimal contamination. There is significant contamination of the operator during ultrasonic scaling and air polishing and appropriate personal protective equipment should be used for these procedures with particular attention to respiratory, facial and body protection. A few studies showed that some droplet contamination took between 30 min and 1 h to settle. Consideration should be given to the use of lower power settings to reduce the amount and spread of contamination.

## Supplementary information

Appendix 1: Quality Assessment

Appendix 2: Equipment Table

Appendix 3: Person Contamination Table

Appendix 4: Breakdown of Studies

Appendix 5: Distances of Contamination 
