# Supplementary material for: Dental periodontal procedures: a systematic review of contamination (splatter, droplets and aerosol) in relation to COVID-19
Source: BDJ Open. 2021 Mar 24;7:15. doi: 10.1038/s41405-021-00070-9 (PMC7988384; doi:10.1038/s41405-021-00070-9)
Supplement: Supplementary file 1 — Appendix 1: Quality Assessment [file 41405_2021_70_MOESM1_ESM.docx]

**Appendix 1**

**Quality Assessments**

|  | Study ID | Perio-related studies (n=50) | **Was the study industry funded (related to the study materials being investigated) ?** | **Was there a conflict of interest?** | **Relevance to routine clinical dentistry** | **Procedure description** | **Equipment used reporting** | **Sample size** | **Controls (for microbial studies?)** | **Outcome (Contamination)** | **Overall Microbiological Sensitivity Assessment (Bacterial)** |
| --- | --- | --- | --- | --- | --- | --- | --- | --- | --- | --- | --- |
|  |  |  |  |  |  |  |  |  |  |  |  |
| **Ultrasonic studies n=44** | 5 | Balcos et al. 2019 | Amber | Amber | Green | Red | Amber | Red | N/A | Amber | N/A |
|  | 6 | Barnes et al. 1998 | Amber | Amber | Green | Amber | Amber | Red | N/A | Amber | N/A |
|  | 8 | Bentley et al. 1994 | Red | Amber | Red | Amber | Red | Red | Green | Amber | Low |
|  | 9 | Choi et al. 2018 | Green | Green | Green | Amber | Red | Red | Red | Amber | Low |
|  | 10 | Chuang et al. 2014 | Amber | Green | Green | Amber | Green | Red | Green | Red | Low |
|  | 16 | Devker et al. 2012 | Amber | Amber | Green | Amber | Red | Red | Green | Red | Low |
|  | 21 | Feres et al. 2010 | Red | Red | Green | Red | Red | Red | Green | Red | Medium |
|  | 22 | Fine et al. 1992 | Red | Amber | Green | Red | Amber | Red | Green | Red | N/A |
|  | 23 | Fine et al. 1993 | Red | Red | Red | Red | Red | Red | Red | Red | Low |
|  | 24 | Fine et al. 1993 | Red | Red | Green | Red | Red | Red | Green | Amber | Low |
|  | 25 | Graetz et al. 2014 | Amber | Amber | Red | Amber | Green | Red | N/A | Amber | N/A |
|  | 29 | Greenier 1995 | Amber | Amber | Green | Amber | Red | Red | Green | Amber | High |
|  | 31 | Gupta et al. 2014 | Amber | Green | Green | Amber | Red | Red | Green | Amber | Low |
|  | 32 | Hallier et al. 2010 | Amber | Red | Green | Red | Red | Red | Green | Red | Low |
|  | 33 | Harrel et al. 1996 | Amber | Green | Red | Amber | Green | Red | N/A | Amber | N/A |
|  | 34 | Harrel et al. 1998 | Amber | Amber | Red | Amber | Green | Red | N/A | Amber | N/A |
|  | 37 | Holloman et al. 2015 | Red | Amber | Green | Amber | Green | Red | Green | Green | High |
|  | 41 | Jawade et al. 2016 | Amber | Green | Green | Amber | Amber | Red | Green | Red | Low |
|  | 44 | Kaur et al. 2014 | Amber | Green | Green | Red | Red | Red | Green | Amber | Low |
|  | 45 | King et al. 1997 | Amber | Amber | Green | Green | Amber | Red | Red | Amber | Low |
|  | 48 | Labaf et al. 2011 | Amber | Amber | Green | Amber | Red | Red | Red | Amber | Low |
|  | 90 | Miller et al. 1971 | Amber | Amber | Green | Green | Amber | Red | Green | Amber | Low |
|  | 52 | Mohan and Jagannathan 2016 | Amber | Green | Green | Red | Red | Green | Green | Green | Low |
|  | 54 | Narayana et al. 2016 | Amber | Green | Green | Red | Red | Green | Red | Red | Low |
|  | 55 | Neiatidanesh et al. 2013 | Green | Green | Green | Amber | Amber | Red | N/A | Amber | N/A |
|  | 59 | Prospero 2003 | Red | Red | Red | Amber | Red | Red | Red | Amber | Low (total counts) Low (for selective media) |
|  | 60 | Purohit et al. 2010 | Amber | Amber | Green | Amber | Amber | Red | Green | Amber | Low |
|  | 61 | Ramesh et al. 2015 | Amber | Green | Green | Amber | Amber | Red | Red | Amber | Low |
|  | 62 | Rao et al. 2015 | Amber | Amber | Green | Red | Red | Red | Red | Red | Low |
|  | 64 | Reddy et al. 2012 | Amber | Green | Green | Red | Red | Red | Green | Red | Low |
|  | 65 | Retamal-Valdes et al. 2017 | Red | Amber | Green | Green | Amber | Red | Green | Amber | Medium |
|  | 66 | Rivera-Hidalho et al. 1999 | Amber | Amber | Red | Amber | Green | Red | N/A | Amber | N/A |
|  | 69 | Sadun et al. 2020 | Red | Red | Red | Red | Red | Red | Red | Amber | Low |
|  | 70 | Saini 2015 | Green | Green | Green | Green | Amber | Red | Green | Amber | Low |
|  | 71 | Sawhney et al. 2015 | Green | Green | Green | Green | Red | Red | Green | Amber | Low |
|  | 72 | Serban et al. 2013 | Amber | Amber | Green | Red | Red | Red | Green | Amber | Low |
|  | 73 | Sethi et al. 2019 | Amber | Green | Green | Amber | Red | Red | N/A | Amber | Low |
|  | 74 | Shetty et al. 2013 | Amber | Green | Green | Amber | Red | Red | Red | Amber | Low |
|  | 76 | Singh et al. 2016 | Green | Green | Green | Amber | Amber | Red | Green | Amber | Low |
|  | 78 | Swaminathan et al. 2014 | Amber | Amber | Green | Amber | Green | Red | Red | Amber | Low |
|  | 80 | Timmerman et al. 2004 | Amber | Amber | Green | Amber | Green | Amber | Green | Amber | High |
|  | 83 | Veena et al. 2015 | Green | Green | Red | Amber | Green | Red | N/A | Amber | N/A |
|  | 85 | Watanabe et al. 2013 | Green | Green | Green | Red | Amber | Red | Red | Red | N/A |
|  | 87 | Yamada et al. 2011 | Amber | Amber | Green | Amber | Red | Red | N/A | Amber | N/A |
| **Air polishing n=4** | 35 | Harrel et al. 1999 | Amber | Green | Red | Green | Green | Red | N/A | Amber | Medium |
|  | 18 | Dos Santos 2014 | Amber | Green | Green | Green | Amber | Green | Green | Amber | Low |
|  | 49 | Logothetis 1995 | Green | Amber | Green | Green | Red | Red | Green | Green | Low |
|  | 53 | Muzzin 1999 | Red | Amber | Amber | Amber | Red | Red | Green | Red | Low |
| **Hand scaling n=3** | 34 | Harrel et al. 1998 *(also in US) | Amber | Amber | Red | Amber | Green | Red | N/A | Amber | N/A |
|  | 89 | Micik 1969 | Amber | Amber | Amber | Amber | Amber | Red | Green | Amber | Low |
|  | 63 | Rautemma 2006 | Amber | Amber | Green | Red | Red | Red | Green | Red | Low |
| **Prophylaxis studies n=2** | 89 | Micik 1969* (also in HS) | Amber | Amber | Amber | Amber | Amber | Red | Green | Amber | Low |
|  | 90 | Miller et al. 1971* (also in US) | Amber | Amber | Green | Green | Amber | Red | Green | Amber | Low |
