# Supplementary material for: Dental periodontal procedures: a systematic review of contamination (splatter, droplets and aerosol) in relation to COVID-19
Source: BDJ Open. 2021 Mar 24;7:15. doi: 10.1038/s41405-021-00070-9 (PMC7988384; doi:10.1038/s41405-021-00070-9)
Supplement: Supplementary file 2 — Appendix 2: Equipment Table [file 41405_2021_70_MOESM2_ESM.docx]

**Appendix 2**

**Equipment table**

|  | Study ID | Author | Primary equipment model detail | Speed/Power | Baseline suction? | Comparative study? | Procedure Duration | Additional Information |
| --- | --- | --- | --- | --- | --- | --- | --- | --- |
| Ultrasonic n=44 | 5 | Balcos et al. 2019 | Woodpecker UDS-K piezoelectric ultrasonic scaler | 3 frequency levels (3-5) tested | **YES**  Simple or surgical suction (comparison) | Simple v surgical suction  3 frequencies tested | 10 min | Comparison of simple or surgical suction testing 3 ultrasonic frequency levels |
|  | 6 | Barnes et al. 1998 | magnetostrictive ultrasonic scaler | 25,000 cycle per second | **YES**  Large diameter (8x10mm) disposable suction tip (HVE) (3-5cm away from operating site). attached to the central evacuation unit to capture aerosols | No | 30 Seconds |  |
|  | 8 | Bentley et al. 1994 | NOT STATED | NOT STATED | **YES**  Conventional saliva ejector | No | 30 minutes (aerosol with ultrasonic) |  |
|  | 9 | Choi et al. 2018 | NOT STATED | NOT STATED | **NOT STATED** | Mouthrinse -gargling with and without 0.01% chlorhexidine before US scaling | not stated |  |
|  | 10 | Chuang et al. 2014 | Bobcat Cavitron, Dentsply | 25 kHz power (supplied with distilled water) | **YES**  Regular-power fluent suction (low volume) | No | 30 sec gargling  Scaling time: approx 15 min |  |
|  | 16 | Devker et al. 2012 | Piezoelectric with ultrasonic inserts [Varios (550 NSK Japan)] | NOT STATED | **YES**  **Low volume suction**  **High volume suction**  - HIGH VOLUME EVACUATOR COMPARISON WITH & WITHOUT HVE (vacuum of HVE attachment standardised at 140 mm Hg) | High Volume Evacuator (HVE) with and without suction  3 grps: (i) Mouthrinse -  Rinse of 0.2% chlorhexidine gluconate prior to scaling.  (ii): HVE attachment during scaling  (iii): Mouthrinse -  Rinse of 0.2% chlorhexidine gluconate prior to scaling & use of HVE attachment. Control grp: split-mouth design No suction or mouth rinse and Mouthrinse and HVE alone or together | 10 min | Comparison with and without HVE suction |
|  | 21 | Feres et al. 2010 | Cavitron Select, Dentsply with distilled water | NOT STATED | **NOT STATED** | Mouthrinse:  (i) 0.05% CPC  (ii) 0.12% CHX  (iii)water  (iv) no rinse | 10 min | N/A |
|  | 22 | Fine et al. 1992 | Cavitron Model 3000, Dentsply International, York, PA. | Medium setting | **NOT STATED** | Mouthrinse:  (i) antiseptic  (ii) 5% hydroalcohol control rinse | 10 min | mouthwash split mouth |
|  | 23 | Fine et al. 1993 | Cavitron Model 3000, Dentsply | NOT STATED | **NOT STATED** | No | ultrasonic scaling **5 min** (hand scaling 30 min) | Air flow vacuum set at 55 cubic feet/hour. |
|  | 24 | Fine et al. 1993 | **MODEL DETAILS**: NOT STATED | **SPEED/POWER**: NOT STATED | **NOT STATED** | Control v antiseptic mouthrinse | 10 min | N/A |
|  | 25 | Graetz et al. 2014 | 3 devices 1) **sonic scaler** AIR (Synea, W&H, Bürmoos, Austria) and 2). T**wo ultrasonic hand-pieces ***TIG (Tigon+, W&H, Bürmoos, Austria) and *VEC (Vector, Dürr, Bietigheim-Bissingen, Germany) with slimline tips | NOT STATED | **YES**  Saliva ejector and high-speed evacuation  (standardized suction of 300 S ml/min and a depression of 180 mbar) | Compared high volume cannula tips | 2 min | 1st arm: saliva ejector; 2nd arm: high-speed evacuation system |
|  | 29 | Greenier 1995 | **MODEL DETAILS**: NOT STATED | NOT STATED | **NOT STATED** | No | Ultrasonic scaling=15 min |  |
|  | 31 | Gupta et al. 2014 | **MODEL DETAILS**: Piezoelectric | Not stated | **YES**  MOTORIZED SUCTION | YES  3 GROUPS - mouthrinse a) HRB; 2) 0.2% chlorhexidine gluconate (CHX) and 3) water | 30 min | High volume suction |
|  | 32 | Hallier et al. 2010 | **MODEL DETAILS**: NOT STATED | NOT STATED | **YES**  HIGH VOLUME SUCTION | YES  Compares with different procedures - cavity prep, H&E and tooth extraction | NOT STATED | Suction? HVA |
|  | 33 | Harrel et al. 1996 | **MODEL DETAILS**: NOT STATED | Scaler set at full power from 3 settings | **YES**  COMPARISON with and without High Volume Evacuator attachment (used at 140mm/Hg ) | With and without High Volume Evacuator attachment | 1 min | water coolant volume 17.5ml per min |
|  | 34 | Harrel et al. 1998 | **MODEL DETAILS**  x4 US scalers - 1)Autotuned Magnetostrictive 25,000-Hz, 2)Autotuned Magnetostrictive 30,000-Hz , 3)Manually Tuned Magnetostrictive 25,000-Hz and 4)Autotuned Piezoelectric 42,000-Hz.  Tested with variety of INSERTS and CONTROL Gracey 1/2 hand curette | **SPEED/POWER**: inserts tested at high, med and low and 2 power settings for manually tuned unit -tuned/detuned | **NO** | Comparison of ultrasonic power levels and tip | 3 sec | No coolant water |
|  | 37 | Holloman et al. 2015 | **MODEL DETAILS** 30kh Cavitron Select SPS with Dentsply 30k slimline scaling tip | water dispensed & power settings -50% power 50% lavage. | **YES -**  LOW VOLUME SUCTION | YES  LOW VOLUME SUCTION v LOW VOLUME SUCTION WITH IOSLITE DEVICE | Mean (SD) Times scaling- Control 10.08 (2.75) & Test 9.92 (2.25) | suction - set on high & low |
|  | 41 | Jawade et al. 2016 | **MODEL DETAILS**: NS (universal tip) | NOT STATED | **YES**  - HIGH VOLUME SUCTION ALL | Ultrasonic Coolant-  (i) Distilled water  (ii) Povidone iodine (iii) Chlorhexidine | 20 minutes | N/A |
|  | 44 | Kaur et al. 2014 | **MODEL DETAILS**: NOT STATED ultrasonic scaler (universal tip) | NOT STATED | **YES**  - SALIVA EJECTOR FOR ALL | Mouthrinse-  (i) 0.2% Chlorhexidine  (ii) Povidone iodine  (iii) irrigation Ozone | 10 minutes | Saliva ejector |
|  | 45 | King et al. 1997 | **MODEL DETAILS**: magnetostrictive ultrasonic scaler Cavitron Model 3000, Dentsply & univeral insert | NOT STATED | **YES**  - WITH AND WITHOUT HIGH VOLUME SUCTION TIP (disposable high-volume suction tube attached to the handle of the ultrasonic scaler and surrounded the tip of the insert) | WITH AND WITHOUT HIGH VOLUME SUCTION TIP (aerosol reduction device) | 5 minutes | NA |
|  | 48 | Labaf et al. 2011 | **MODEL DETAILS**: Cavitron (Dentsply, USA) | Not stated | **NOT STATED** | Compares endodontic, prosdthodontic and periodontic treatments CFUs | 3 hours | NA |
|  | 90 | Miller et al. 1971 | **MODEL DETAILS**: Cavitron Ultrasonics Inc ultrasonic curette | NOT STATED | **NOT STATED** | No | 30 sec |  |
|  | 52 | Mohan and Jagannathan 2016 | **MODEL DETAILS**:NOT STATED  **SPEED/POWER**: NOT STATED |  | **NOT STATED** | Preprocedural Mouthrinse - 0.2% chlorhexidine v Saline | NOT STATED | N/A |
|  | 54 | Narayana et al. 2016 | **MODEL DETAILS**: EMS ultrasonic scaler | NOT STATED | **YES**  - COMPARISON WITHOUT and WITH high volume evacuation ran at 30–40 psi kg/cm^2^. | Mouthrinse of chlorhexidine 0.12% alone  With HVE & rinse v without HVE & rinse  And without HVE or mouthrinse | NOT STATED | High volume evacuation |
|  | 55 | Neiatidanesh et al. 2013 | **MODEL DETAILS**: Cavitron, Dentsply, Addlestone, UK | NOT STATED | **NOT STATED** | Compares splatter generated by Periodontal and Prosthetic procedures | 44 min (average duration of the procedure) | N/A |
|  | 59 | Prospero 2003 | **MODEL DETAILS**: NOT STATED | NOT STATED | **NOT STATED** | No | NOT STATED | N/A |
|  | 60 | Purohit et al. 2010 | **MODEL DETAILS:** Magnetostrictive scaler | 30 kHz, with a water pressure of 0.3 MPa during each treatment. | **NOT STATED** | Mouthrinse - with chlorhexidine 0.12% v without (water rinse) | NOT STATED | N/A |
|  | 61 | Ramesh et al. 2015 | **MODEL DETAILS**: Piezoelectric scaler unit | NOT STATED | **YES**  - HIGH VOLUME SUCTION ALL | Mouthrinse - with chlorhexidine 0.12% v without (saline). Grp3 - topical solution of 1.5% hydrogen peroxide pre CHX rinse | 5 min | High vacuum suction |
|  | 62 | Rao et al. 2015 | **MODEL DETAILS**: Piezoelectric | NOT STATED | **YES**  - Motorized suction ALL | Mouthrinse - chlorhexidine 0.2% v without rinse | 30 min | Motorized suction |
|  | 64 | Reddy et al. 2012 | **MODEL DETAILS**: NOT STATED | NOT STATED | **NOT STATED** | Mouthrinse - (3 groups) (1) tempered chlorhexidine 0.2% v (2) non-tempered chlorhexidine 0.2% v (3) sterile water | NOT STATED | N/A |
|  | 65 | Retamal-Valdes et al. 2017 | **MODEL DETAILS**: Cavitron Select, Dentsply, York, Pa | Frequency of 25 kHz on less than 50% power | **NOT STATED** | Mouthrinse - (i) 0.075% CPC, 0.28% Zn, 0.05% F rinse v (ii) Water v (iii) chlorhexidine 0.2% v (iv)  no rinsing | 10 min | N/A |
|  | 66 | Rivera-Hidalho et al. 1999 | **MODEL DETAILS**: (Cavitron 3000)+ (S) design insert (Through flow insert TFI-10, Dentsply Preventive Care)+(F) design insert (Focused spray insert FSI-10, Dentsply Preventive Care, York, PA). | 30,000 cycle per second set on max. power | **YES/NO**  - WITH & WITHOUT AEROSOL REDUCTION DEVICE (ARD) | WITH & WITHOUT AEROSOL REDUCTION DEVICE for x2 inserts -focused spray and standard ultrasonic insert | 1 min | N/A |
|  | 69 | Sadun et al. 2020 | **MODEL DETAILS**: NOT STATED | **SPEED/POWER**: NOT STATED | **NOT STATED** | Mouthrinse -20 mL of Listerine v dyed sterile water (control) | NOT STATED | N/A |
|  | 70 | Saini 2015 | **MODEL DETAILS**: EMS ultrasonic scaler | **SPEED/POWER**: Coolant water flow and power settings adjusted to a medium mode and coolant water flow volume adjusted to 15 ml per min | **NOT STATED** | Mouthrinse-  (i) Chlorine Dioxide CI0_2_  (ii) water  (iii) 0.2% CHX | 10 min | N/A |
|  | 71 | Sawhney et al. 2015 | **MODEL DETAILS**: NOT STATED | **SPEED/POWER**: Medium power setting and medium water pressure | **YES/NO**  - WITH AND WITHOUT HIGH SUCTION DEVICE | SPLIT-MOUTH DESIGN  WITH AND WITHOUT HIGH SUCTION DEVICE  Mouthrinse-  (i) Water  (ii) Chlorine Dioxide CI0_2_  (iii) Listerine | NOT STATED | High volume evacuation |
|  | 72 | Serban et al. 2013 | **MODEL DETAILS**: NOT STATED | **SPEED/POWER**: NOT STATED | **NOT STATED** | Mouthrinse-  (i) Sterile Water  (ii) 0.1% Chlorhexidine | NOT STATED |  |
|  | 73 | Sethi et al. 2019 | **MODEL DETAILS:** NOT STATED | **SPEED/POWER**: NOT STATED | **YES**  - SALIVA EJECTOR ALL | Ultrasoninc coolant-  (i) Chlorhexidine  (ii) Cinamon extract  (iii) Distilled water | 20 min |  |
|  | 74 | Shetty et al. 2013 | **MODEL DETAILS**: NOT STATED | **SPEED/POWER**: NOT STATED | **NOT STATED** | Mouthrinse-  (i) Water  (ii) 0.2% Chlorhexidine  (iii) Tea tree oil | 10 min | N/A |
|  | 76 | Singh et al. 2016 | **MODEL DETAILS**: Piezoelectric ultrasonic unit | **SPEED/POWER**: 'Constant' frequency and 'constant' water coolant pressure | **YES**  - motorized suction ALL | No | 20 min | Motorized suction |
|  | 78 | Swaminathan et al. 2014 | **MODEL DETAILS:** Piezoelectric ultrasonic unit. | **SPEED/POWER:** Unit set at 7 KiloHertz for all patients | **YES**  - High speed evacuator/ suction used for ALL. | Mouthrinse-  (i) Saline  (ii) 0.2% Chlorhexidine  (iii) Herbal | 30 min | N/A |
|  | 80 | Timmerman et al. 2004 | **MODEL DETAILS:** Piezoelectric ultrasonic scaler (Piezo Master 400, EMS, Nyon, Switzerland) with an exchangeable 250 ml coolant reservoir | **SPEED/POWER:** NOT STATED | **YES**  - either high-volume evacuation (HVE) canula of 8.0mm in diameter suction flow of 6.0 l/min for HVE **or** conventional dental suction (CDS) canula of 3.3mm in diameter with a suction flow of 1.1 l/ min for CDS | High volume evacuation (HVE) & conventional dental suction (CDS) | 40 min | The use of the HVE and the CDS was randomly assigned within each patient. For both types of suction, regular, commercially available, disposable tubes were used: a canula of 8.0mm in diameter with a suction flow of 6.0 l/min for HVE and a canula, 3.3mm in diameter with a suction flow of 1.1 l/ min for CDS (Fig. 2). The HVE was handled by an assistant. |
|  | 83 | Veena et al. 2015 | **MODEL DETAILS:** Autotuned Magnetostrictive ultrasonic scaler | **SPEED/POWER:** 25,000 Hz/ | **YES**  - SIMULTANEOUS USE OF A CONVENTIONAL LOW VOLUME SALIVA EJECTOR | No | 15 min | low volume saliva ejector |
|  | 85 | Watanabe et al. 2013 | **MODEL DETAILS:** Solfy F, Morita Mfg. Corp,. Tokyo | **SPEED/POWER:** NOT STATED | **NOT STATED** | No | NOT STATED | N/A |
|  | 87 | Yamada et al. 2011 | **MODEL DETAILS**: NOT STATED | **SPEED/POWER**: NOT STATED | **NOT STATED** | PROCEDURES 3RD MOLAR EXT, FULL CROWN PREP, INLAY CAVITY PREP AND ULTRASONIC SCALING | NOT STATED | Extraoral evacuator systems (2 at different distances 50cm, 100cm) |
