# Supplementary material for: Dental periodontal procedures: a systematic review of contamination (splatter, droplets and aerosol) in relation to COVID-19
Source: BDJ Open. 2021 Mar 24;7:15. doi: 10.1038/s41405-021-00070-9 (PMC7988384; doi:10.1038/s41405-021-00070-9)
Supplement: Supplementary file 3 — Appendix 3: Person Contamination Table [file 41405_2021_70_MOESM3_ESM.docx]

**Appendix3**

**Person Contamination Table**

| **Person Contamination Table** | | | | | | |
| --- | --- | --- | --- | --- | --- | --- |
| **Periodontal Papers n=44** | | | | | | |
|  | **ID** | **Author** | **Person contamination?** | **OPERATOR** | **ASSISTANT** | **PATIENT** |
| **1** | **5** | Balcos et al. 2019 | YES | **Body:** Visible dye splatter size using simple suction was between 12.5cms (low frequency) to 25.4cms surface area (high frequency) |  |  |
| **2** | **8** | Bentley et al. 1994 | YES | **Face:** mask 77 (4) **Head** 36 (6) **Body**: Chest 25 (118) |  | **Body:** Chest 242 (0) |
| **3** | **9** | Choi et al. 2018 | YES | **Face:** Glasses 52.50 ± 4.95 CFU |  |  |
| **4** | **10** | Chuang et al. 2014 | YES | **Body:** Collar inside full face shield 34 +/- 12CFU (case A) |  |  |
| **5** | **16** | Devker et al. 2012 | YES | **Face**: Nose 90.83 CFU | **Face:** Nose 88.36 CFU | **Body:** Chest 107.13 CFU |
| **7** | **21** | Feres et al. 2010 | YES | **Face:** Forehead 73 (38) CFU |  | **Body:** Chest 66 (412) CFU |
| **8** | **25** | Graetz et al. 2014 | YES | Head and Body: Spatter contamination on mannekin head - fluorescein  solution photographic images |  | **Head:** Spatter contamination on mannekin head - fluorescein solution photographic images |
| **9** | **31** | Gupta et al. 2014 | YES | **Body:** Chest 93.125 ± 9.61 | **Body:** Chest 26.125 ± 6.03 | **Body:** Chest 280.625 ± 22.43 |
| **10** | **44** | Kaur et al. 2014 | YES | **Face:** Mask (3 groups)= 28.7± 18.6; 35.2± 24.4; 43.8±37.2 CFU |  |  |
|  | **45** | King et al. 1997 | YES | **Face:** Face-shield 1.22±1.53 CFU |  | **Face:** 45.13 ± 28.95 CFU |
| **11** | **55** | Neiatidanesh et al. 2013 | YES | **Face:** Face-shield contamination by visible groups of splashes recorded (nose, mouth eyes)  Mean facial splashes (SD)- outer corner of eye: 8.82 (9.79), Middle of eye 8.06 (10.73), eye inner corner 10.65 (13.9), cheek 7.01 (6.17); lip commissures 8.71 (9.68); lateral ala 10.04 (11.56); middle of lips 7.94 (7.54) TOTAL 9.84 (7.68) *P*=0.013 |  |  |
| **12** | **59** | Prospero 2003 | YES | **Face:** Mask 0.0676 CFU cm2/min |  |  |
| **13** | **60** | Purohit et al. 2010 | YES | **Body:** Chest 72.4 mean CFU (SD 5.7) |  | **Body:** Chest 102.4 mean CFU (SD 4.5) |
| **14** | **61** | Ramesh et al. 2015 | YES | **Body:** Side 12.80 CFU | **Body:** Side 11.80 CFU | **Body:** Chest 12.60 CFU |
| **15** | **62** | Rao et al. 2015 | YES | **Body:** Chest range 7-10 CFU |  | **Body:** Chest range 5-6 CFU |
| **16** | **65** | Retamal-Valdes et al. 2017 | YES | **Face:** Forehead 94 CFU |  | **Body:** Chest= 60 CFU |
| **17** | **70** | Saini 2015 | YES |  |  | **Body:** Chest= 90.6(±2.84) CFU |
| **18** | **71** | Sawhney et al. 2015 | YES |  |  | **Body:** Chest (results combined for 3 locations - chest, 6 inches from mouth & each side of Pt's chair - Mean CFU 16; 80% |
| **19** | **72** | Serban et al. 2013 | YES | **Face:** Mask (M = 121.35 CFU/m^3^) |  |  |
| **20** | **73** | Sethi et al. 2019 | YES |  |  | **Body:** Chest, right side, and left side of the patient =1396.15 ± 214.93, 1064.05 ± 26.69 |
| **21** | **83** | Veena et al. 2015 | YES | **Face:** Mask (inside) 4, **Body:** Head 10, Chest 58 & R.arm 88, L.arm 22 cm^2^  Contamination (dye) identified by cm^2^ | **Face:** Mask (inside) 1**, Body:** Head 12, Chest 34 & R.arm 15, L.arm 42 cm^2^  Contamination (dye) identified by cm^2^ |  |
| **22** | **85** | Watanabe et al. 2013 | YES | **Face:** Mask (median 672.0, IQR 448.0-938.6), dental goggles (median 1106.8, IQR 657.4-1580.3)  **Body:** Chest (median 672.0, IQR 448.0-938.6) and Right arm (median 761.0, IQR 670.8-914.3) |  | **Face:** Dental goggles (median 1519.5, IQR 913.5-1866.7) |
| **Air Polishing Papers n=3** | | | | | | |
|  | **ID** | **Author** | **Person contamination?** | **OPERATOR** | **ASSISTANT** | **PATIENT** |
| **1** | **18** | Dos Santos 2014 | YES | **Face:** Forehead & Chest (10cm down from mouth) mean CFU 7.16x10^2^ (p=0.0051) sd= 351.03 |  | **Body:** Chest (thoratic region 15cm from mouth) mean CFU 1.68x10^3^ (p=0.0035) sd = 953.80 |
| **3** | **49** | Logothetis 1995 | YES | **Face: mask 82.8 (±12.8) - 84.5 (± 9.9) mean CFU** |  |  |
| **4** | **53** | Muzzin 1999 | YES | **Face: mask 40.9 ± 33.80 CFUs** |  | **Body: Chest 148.00 ± 145.00 CFUs** |
| **Hand scaling papers n=1** | | | | | | |
|  | **ID** | **Author** | **Person contamination?** | **OPERATOR** | **ASSISTANT** | **PATIENT** |
| **3** | **63** | **Rautemma 2006** | YES | **Face: mask swabbed but data not reported** | **Face: mask swabbed but data nor reported** |  |
