# Supplementary material for: Dental periodontal procedures: a systematic review of contamination (splatter, droplets and aerosol) in relation to COVID-19
Source: BDJ Open. 2021 Mar 24;7:15. doi: 10.1038/s41405-021-00070-9 (PMC7988384; doi:10.1038/s41405-021-00070-9)
Supplement: Supplementary file 5 — Appendix 5: Distances of Contamination [file 41405_2021_70_MOESM5_ESM.docx]

**Appendix 5**

**Distances of contamination**

**Table for Droplets Under 1m only**

|  |  |  |  |  | Study | Measure | 0-49cm | 50-99cm |
| --- | --- | --- | --- | --- | --- | --- | --- | --- |
| 1 | Bentley 1994 | | | | CFU /cm | 12-78 (30.4 cm) | 23-69 (60.9) |  |
| 2 | Devker 2012 | | | | CFU/cm |  | 22.36 (91cm) |  |
| 3 | Feres 2010 | | | | CFU/cm | 79 (15cm) |  |  |
| 4 | Harrel 1996 | | | | Contaminated squares | 132.2 (SD ± 68) |  |  |
| 5 | Holloman 2015 | | | | CFU | during 3.61 (0.95) (0.74-3.21) after 2.00 (1.17) (0.91-1.52) |  |  |
| 6 | King 1997 | | | | CFU | 45.13 ±28.9 |  |  |
| 7 | Mohan 2016 | | | | CFU |  | 891.1 ± 595.19 |  |
| 8 | Narayana 2016 | | | | CFU | 100.73 (SD 89.34) |  |  |
| 9 | Ramesh 2015 | | | | CFU |  | Assistant side 11.80  Operator side = 12.80 |  |
| 10 | Rivera Hidalho 1999 | | | | Contaminated squares | 98.5 contaminated squares |  |  |
| 11 | Sawhney 2015 | | | | CFU as % | 16 (80%) |  |  |
| 12 | Sethi, 2019 | | | | CFU | Chest, right side, and left side of the patients were 1396.0 ± 214.93, 1064.05 ± 26.69, and 1009.85 ± 23.29 (mean ± SD), respectively" |  |  |
| 13 | Shetty 2013 | | | | CFU | Operator’s nose level 120.85 Dental assistant’s nose level and 50.75 |  |  |
| 14 | Singh 2016 | | | | CFU | 7.869+/- 0.862 |  |  |
| 15 | Swaminathan 2014 | | | | Mean rank of CFU reported | 30cm= 21.25 | 60cm=20.05 |  |
| 1(16) | Dos Santos 2014 | | | | CFU | 15cm=1.68X10^3^ |  |  |
| 2 (17) | Harrel 1999 | | | | Contaminated squares | 175.29 |  |  |
| 3 (18) | Muzzin 1999 | | | | CFU | 20.1 (sd 53.9) |  |  |

**Table for Droplets Studies Including Measures for Droplets Under 1m and Over 1m**

|  |  |  |  |  | Study | | Measure | | 0-49 | | 50-99 | | | 1m+ | | |
| --- | --- | --- | --- | --- | --- | --- | --- | --- | --- | --- | --- | --- | --- | --- | --- | --- |
| 1 | Chuang 2014 | | | | | CFU/M^3^ | | 10cm above patient 2243±256  30cm above pt 934±178 | | 50 cm horizontally 178 ±51 and 148±122  50cm horizontally at 15 degrees 1129 ±113 and 3432±653 | | | 100cm from patient 141±32 and 138±122  100cm horizontally 1346±145 (left of patient)  150 cm horizontally 43±12 and 33±75  And 124±109 and 1433±131 (front of patient) | |  |  |
| 2 | Jawade 2016 | | | | | CFU/CM | | (124.5 ± 30.08 ) . | | Right side 0.4m (165.3 ±18.47). Left side 0.4m (mean 128); | | 2m behind the patient (mean= 79) | | | |  |
| 3 | Labaf 2011 | | | | | CFU | |  | | Mean CFUs were 124.71 (SD 7.74) CFUs at 50cm | | 42.86 (SD 21.12) CFUs at 150cm, 50.43(SD 24.57) CFUs at 200cm and 722.7(SD13.31) CFUs at 300cm | | | |  |
| 4 | Miller et al. 1971 | | | | | CFU/ft^2^ | | 100-1000 cfu/ft^2^ | | 100-1000 cfu/ft^2^ | | 100-1000 cfu/ft^2^ | | | |  |
| 5 | Saini 2015 | | | | | CFU (mean) | | 1 ft from the reference point (Operator position)= 90.37(±2.72)1 ft from the reference point (Assistant position)= 88.56(±3.36) | | 2 ft from the reference point (12 o’ clock position)= 71.51(±3.30); | | 8 ft from the reference point (6 o’ clock position)= 54.35(±3.13) | | | |  |
| 6 | Timmerman 2004 | | | | | CFU (mean) | | 40cm 0-5 min 2.5 (2.9), 20-25min 1.8 (1.625+mins 4.3 (3.5) | |  | | 150cm 6.3 (5.9),150 cm, 4.0 (4.1), 150 cm 10.3 (9.5) | | | |  |
| 7 | Veena 2015 | | | | | Contaminated 8squares | | 12 o’clock= 50; 2 o’clock= 42; 4 o’clock= 83; 6 o’clock= 72; 8 o’clock= 5; 10 o’clock positions= 21. | | (2 ft): 12 o’clock= nil; 2 o’clock= nil; 4 o’clock= 12; 6 o’clock= nil; 8 o’clock= 2; 10 o’clock=14. | | (4ft)= 2 O'clock= 4 squares were found. | | | |  |
| 8 | Yamada 2011 | | | | | Positive reaction dots | | 33% (11/33) | | 12% (4/33) | | 60% (33/55) | | | |  |
| 1 (9) | Logothetis 1995 | | | | | CFU | |  | | 60.1cm=82.8 (sd 12.8) | | Behind the dental chair (3ft away)= 69.3 (15.9); right hand side to the patient (3 ft away)=56.1 (12.1). Left hand side to the patient= 43.8 (3.7), Another point at the left hand side to the patient (5ft/8 inch)= 34 (3.8). In front of the patient (6 ft and 9ft away)=27.3 (3.9). | | | |  |

**Table for Droplets Studies Including Measures for Droplets Over 1m**

|  |  |  | | Study | | Measure | | 0-49cm | | 50-99cm | 1m+ | | |  |  |
| --- | --- | --- | --- | --- | --- | --- | --- | --- | --- | --- | --- | --- | --- | --- | --- |
| 1 | | | Graetz 2014 | | Contaminated squares | |  | |  | | | (median [25th; 75th percentiles]: 0.18 [0.07; 1.05]) compared to supragingival scaling (0.34 [0.1; 2.24]) (p < 0.001). | | |  |
| 2 | | | Kaur 2014 | | CFU (mean) | |  | |  | | | | 179.1±64.9) 2.7m | | |
| 3 | | | Reddy 2012 | | CFU (mean) | |  | |  | | | | 114.7 (±9.14) 1.2 | | |
